# Impact of Blood Pressure Control on Thromboembolism and Major Hemorrhage in Patients With Nonvalvular Atrial Fibrillation: A Subanalysis of the J‐RHYTHM Registry

**DOI:** 10.1161/JAHA.116.004075

**Published:** 2016-09-12

**Authors:** Eitaro Kodani, Hirotsugu Atarashi, Hiroshi Inoue, Ken Okumura, Takeshi Yamashita, Toshiaki Otsuka, Hirofumi Tomita, Hideki Origasa, the J‐RHYTHM Registry Investigators, M. Sakurai, Y. Kawamura, I. Kubota, Y. Kaneko, K. Matsumoto, S. Ogawa, Y. Aizawa, I. Kodama, E. Watanabe, Y. Koretsune, Y. Okuyama, A. Shimizu, O. Igawa, S. Bando, M. Fukatani, T. Saikawa, A. Chishaki, N. Kato, K. Kanda, J. Kato, H. Obata, M. Aoki, H. Honda, Y. Konta, T. Hatayama, Y. Abe, K. Terata, T. Yagi, A. Ishida, T. Komatsu, H. Tachibana, H. Suzuki, Y. Kamiyama, T. Watanabe, M. Oguma, M. Itoh, O. Hirono, Y. Tsunoda, K. Ikeda, T. Kanaya, K. Sakurai, H. Sukekawa, S. Nakada, T. Itoh, S. Tange, M. Manita, M. Ohta, H. Eguma, R. Kato, Y. Endo, T. Ogino, M. Yamazaki, H. Kanki, M. Uchida, S. Miyanaga, K. Shibayama, N. Toratani, T. Kojima, M. Ichikawa, M. Saito, Y. Umeda, T. Sawanobori, H. Sohara, S. Okubo, T. Okubo, T. Tokunaga, O. Kuboyama, H. Ito, Y. Kitahara, K. Sagara, T. Satoh, K. Sugi, Y. Kobayashi, Y. Higashi, T. Katoh, Y. Hirayama, N. Matsumoto, M. Takano, T. Ikeda, S. Yusu, S. Niwano, Y. Nakazato, Y. Kawano, M. Sumiyoshi, N. Hagiwara, K. Murasaki, H. Mitamura, S. Nakagawa, K. Okishige, K. Azegami, H. Aoyagi, K. Sugiyama, M. Nishizaki, N. Yamawake, I. Watanabe, K. Ohkubo, H. Sakurada, S. Fukamizu, M. Suzuki, W. Nagahori, T. Nakamura, Y. Murakawa, N. Hayami, K. Yoshioka, M. Amino, K. Hirao, A. Yagishita, K. Ajiki, K. Fujiu, Y. Imai, A. Yamashina, T. Ishiyama, M. Sakabe, K. Nishida, H. Asanoi, H. Ueno, J. D. Lee, Y. Mitsuke, H. Furushima, K. Ebe, M. Tagawa, M. Sato, M. Morikawa, K. Yamashiro, K. Takami, T. Ozawa, M. Watarai, M. Yamauchi, H. Kamiya, H. Hirayama, Y. Yoshida, T. Murohara, Y. Inden, H. Osanai, N. Ohte, T. Goto, I. Morishima, T. Yamamoto, E. Fujii, M. Senga, H. Hayashi, T. Urushida, Y. Takada, N. Tsuboi, T. Noda, T. Hirose, T. Onodera, S. Kageyama, T. Osaka, T. Tomita, K. Shimada, M. Nomura, H. Izawa, A. Sugiura, T. Arakawa, K. Kimura, T. Mine, T. Makita, H. Mizuno, A. Kobori, T. Haruna, M. Takagi, N. Tanaka, H. Shimizu, T. Kurita, K. Motoki, N. Takeda, Y. Kijima, M. Ito, A. Nakata, Y. Ueda, A. Hirata, S. Kamakura, K. Satomi, Y. Yamada, Y. Yoshiga, H. Ogawa, M. Kimura, T. Hayano, T. Kinbara, H. Tatsuno, M. Harada, K. F. Kusano, M. Adachi, A. Yano, M. Sawaguchi, J. Yamasaki, T. Matsuura, Y. Tanaka, H. Moritani, T. Maki, S. Okada, M. Takechi, T. Hamada, A. Nishikado, Y. Takagi, I. Matsumoto, T. Soeki, Y. Doi, M. Okawa, H. Seo, S. Kitamura, K. Yamamoto, M. Akizawa, N. Kaname, S. Ando, S. Narita, T. Inou, Y. Fukuizumi, K. Saku, M. Ogawa, Y. Urabe, M. Ikeuchi, S. Harada, H. Yamabe, Y. Imamura, Y. Yamanouchi, K. Sadamatsu, K. Yoshida, T. Kubota, N. Takahashi, N. Makino, Y. Higuchi, T. Ooie, T. Iwao, K. Kitamura, T. Imamura, K. Maemura, N. Komiya, M. Hayano, H. Yoshida, K. Kumagai

**Affiliations:** ^1^ Department of Internal Medicine and CardiologyNippon Medical SchoolTama‐Nagayama HospitalTokyoJapan; ^2^ Saiseikai Toyama HospitalToyamaJapan; ^3^ Saiseikai Kumamoto HospitalKumamotoJapan; ^4^ The Cardiovascular InstituteTokyoJapan; ^5^ Department of Hygiene and Public HealthNippon Medical SchoolTokyoJapan; ^6^ Department of CardiologyHirosaki University Graduate School of MedicineAomoriJapan; ^7^ Division of Biostatistics and Clinical EpidemiologyUniversity of ToyamaJapan

**Keywords:** anticoagulation, atrial fibrillation, blood pressure, hypertension, thromboembolism, Atrial Fibrillation, Hypertension, High Blood Pressure, Ischemic Stroke, Intracranial Hemorrhage

## Abstract

**Background:**

To clarify the influence of hypertension and blood pressure (BP) control on thromboembolism and major hemorrhage in patients with nonvalvular atrial fibrillation, a post hoc analysis of the J‐RHYTHM Registry was performed.

**Methods and Results:**

A consecutive series of outpatients with atrial fibrillation was enrolled from 158 institutions. Of 7937 patients, 7406 with nonvalvular atrial fibrillation (70.8% men, 69.8±10.0 years) were followed for 2 years or until an event occurred. Hypertension was defined as a systolic BP ≥140 mm Hg, a diastolic BP ≥90 mm Hg, a history of hypertension, and/or antihypertensive drug use. Hypertension was an independent risk factor for major hemorrhage (hazard ratio 1.52, 95% CI 1.05–2.21, *P*=0.027) but not for thromboembolism (hazard ratio 1.05, 95% CI 0.73–1.52, *P*=0.787). When patients were divided into quartiles according to their systolic BP at the time closest to the event or at the end of follow‐up (Q1, <114; Q2, 114–125; Q3, 126–135; and Q4, ≥136 mm Hg), odds ratios for both events were significantly higher in Q4 than in Q1 (thromboembolism, odds ratio 2.88, 95% CI 1.75–4.74, *P*<0.001; major hemorrhage, odds ratio 1.61, 95% CI 1.02–2.53, *P*=0.041) after adjustment for components of CHA_2_ DS_2_‐VASc score, warfarin use, and antiplatelet use. A systolic BP of ≥136 mm Hg was an independent risk factor for thromboembolism and major hemorrhage.

**Conclusions:**

BP control appears to be more important than a history of hypertension and baseline BP values at preventing thromboembolism and major hemorrhage in patients with nonvalvular atrial fibrillation.

**Clinical Trial Registration:**

URL: http://www.umin.ac.jp/ctr. Unique identifier: UMIN000001569.

## Introduction

Atrial fibrillation (AF) is a common arrhythmia and a potent risk factor for cardiogenic embolism.^1^, ^2^ Hypertension is also a common disease and the most frequent comorbidity in patients with AF.^3^, ^4^ Since hypertension is one of the risk factors for thromboembolism and hemorrhagic complications in patients with AF, it is a component of the CHADS_2_ (congestive heart failure, hypertension, age ≥75 years, diabetes mellitus, and history of stroke or transient ischemic attack [TIA]),^5^ the CHA_2_DS_2_‐VASc (CHADS_2_ components, plus vascular disease [coronary artery disease], age 65–74 years, and female sex),^6^ and the HAS‐BLED (hypertension, abnormal renal/liver function, stroke, bleeding history or predisposition, labile international normalized ratio [INR], elderly [>65 years], drugs/alcohol concomitantly)^7^ scores. However, hypertension is not always detected as an independent predictor of thromboembolism or hemorrhagic complications.^8^, ^9^, ^10^, ^11^, ^12^ In patients with nonvalvular AF (NVAF) who were not receiving anticoagulation therapy, hypertension was an independent risk factor for ischemic stroke.^13^ By contrast, the presence of hypertension at baseline was not a significant risk factor for thromboembolism or major hemorrhage in NVAF patients when including those treated with warfarin.^14^, ^15^ Differences in patient characteristics may have contributed to these inconsistent results. Another possible explanation for the inconsistent results was that blood pressure (BP) control was not considered when a history of hypertension was selected as an explanatory variable for multivariate analysis. The association between BP values and the event incidence has not been elucidated in patients with NVAF in any previous report of the J‐RHYTHM Registry.^14^, ^15^, ^16^, ^17^, ^18^, ^19^, ^20^ Therefore, in order to investigate the influence of BP control rather than hypertension on event rates of thromboembolism and major hemorrhage, a post hoc analysis was performed using quartiles of BP values at the time of enrollment and at the time closest to these events in the J‐RHYTHM Registry.

## Methods

### Study Design of the J‐RHYTHM Registry

The J‐RHYTHM Registry was conducted as a nationwide prospective observational study to investigate the present status of anticoagulation therapy and optimal anticoagulation therapy in Japanese patients with AF.^21^ The study design and baseline patient characteristics have been reported elsewhere.^3^, ^21^ Briefly, the study protocol conformed to the Declaration of Helsinki and was approved by the ethics committee of each participating institution. All participants gave written informed consent at the time of enrollment. A consecutive series of outpatients with AF of any type was enrolled from 158 institutions, regardless of the use of antithrombotic drugs. All drugs, including antihypertensive drugs, and their dosages were selected at the discretion of the treating cardiologists. Patients with valvular AF^22^ were excluded from this subanalysis. Seated brachial BP was measured in each patient at the time of enrollment (baseline) and at each follow‐up visit by either the auscultatory method or an automated sphygmomanometer, as was appropriate at each institution. Hypertension was defined as a peripheral systolic BP ≥140 mm Hg, a diastolic BP ≥90 mm Hg,^23^ a prior history of hypertension, and/or the use of antihypertensive drugs.^21^

### Follow‐Up and Definition of End Points

Patients were followed up for 2 years or until an event, whichever occurred first. Thromboembolism included symptomatic ischemic stroke, transient ischemic attack, and systemic embolic events. Major hemorrhage included intracranial hemorrhage (ICH), gastrointestinal hemorrhage, and other hemorrhages requiring hospitalization. If any event occurred during the follow‐up period, BP values at the time prior to and closest to the event were obtained.^21^ The diagnostic criteria for each event have been described elsewhere.^3^, ^21^

### Grouping of Patients

Patients were divided into 2 groups based on the presence of hypertension at the time of enrollment (Hypertension and No‐hypertension) and event incidence rates between the 2 groups were compared. In addition, patients were divided into 4 groups according to the quartiles of their systolic BP at baseline (Q1, <116; Q2, 116–125; Q3, 126–135; and Q4, ≥136 mm Hg) and at the time prior to and closest to an event or at the end of follow‐up (Q1, <114; Q2, 114–125; Q3, 126–135; and Q4, ≥136 mm Hg). Patients were also divided into quartiles based on their diastolic BP.

### Statistical Analysis

Data are presented as mean±SD. The statistical significance of differences in mean values was analyzed using Student *t* test or analysis of variance, as appropriate. Frequencies of parameters or events were compared using the χ^2^ test or Fisher's exact test, as appropriate. Trend among the quartiles was tested by Cochran‐Armitage test for categorical variables or Jonckheere‐Terpstra test for continuous variables, as appropriate. Kaplan–Meier curves between 2 groups were compared with log‐rank tests. A Cox proportional hazards model was used to investigate the influence of hypertension and baseline BP values on events. Odds ratios (ORs) of BP quartiles at the time closest to the event or at the end of the follow‐up period were calculated with multivariate logistic regression analysis. Explanatory variables for multivariate analysis were adopted from well‐known risk factors, i.e., unadjusted model (Model 1) and adjusted models for other components (except hypertension) of CHADS_2_ score (Model 2), other components (except hypertension) of CHA_2_DS_2_‐VASc score (Model 3), and Model 3 plus use of warfarin and antiplatelet (Model 4). The use of antihypertensive drugs was not included as an explanatory variable for multivariate analysis to avoid multicollinearity. Additionally, ORs of BP quartiles for major hemorrhage and ICH were determined after adjustment for other components (except hypertension) of HAS‐BLED score^18^ in 7015 patients with data available for the calculation of this score. In the present analysis, “H” in the HAS‐BLED score was defined as a systolic BP ≥140 mm Hg at the time of enrollment. “L” and “D” were defined as 1 or more episode(s) of INR ≥3.5 during the follow‐up period and concomitant antiplatelet use, respectively.^15^, ^18^ The optimal cutoff BP values for thromboembolic and hemorrhagic events were determined as the points closest to the top‐left corner of the receiver operating characteristic curves (minimum of [1−sensitivity]^2^+[1−specificity]^2^). Two‐tailed *P*‐values of <0.05 were considered to be statistically significant. All statistical analyses were performed with SPSS software version 23.0 (IBM Corporation, Armonk, NY).

## Results

Of 7937 patients with AF enrolled in the J‐RHYTHM Registry,^3^ 421 patients were excluded because they had valvular AF.^22^ Of the remaining 7516 patients with NVAF, 110 (1.5%) patients were lost to follow‐up. Therefore, a total of 7406 patients with NVAF were included in the present analyses.^16^

### Baseline Patient Characteristics and Medications

Baseline patient characteristics and medications of the 2 groups are shown in Table 1. Male sex, cardiomyopathy, and heart failure were less prevalent in the Hypertension group than in the No‐hypertension group. By contrast, mean age, CHADS_2_ score, and the prevalence of diabetes mellitus, use of warfarin or antiplatelet, coadministration of warfarin and antiplatelet, and use of antihypertensive drugs were all higher in the Hypertension group (Table 1). Mean INR and distribution of INR were comparable between the 2 groups, but time in therapeutic range 24 was slightly, but significantly, better in the Hypertension group than in the No‐hypertension group (Table 1). Although 94.8% of patients were taking antihypertensive drugs, baseline BP was higher in the Hypertension group than in the No‐hypertension group (Table 1). Patient characteristics and medications in the quartiles of systolic BP at the time of enrollment are summarized in Table 2. The prevalence of hypertension was 41.2%, 43.1%, 64.8%, and 78.7% in quartiles 1 to 4, respectively (Table 2). Significant trends among quartiles were observed for baseline frequencies of several comorbidities and medications (Table 2).

**Table 1:** Baseline Patient Characteristics and Medications

	Overall	No‐Hypertension	Hypertension	*P‐*Value^a^
Number of patients	7406	2929	4477	
Age, y	69.8±10.0	68.1±10.8	70.8±9.2	<0.001
Sex, male	5241 (70.8)	2146 (73.3)	3095 (69.1)	<0.001
Type of AF				
Paroxysmal	2835 (38.3)	1123 (38.3)	1712 (38.2)	<0.001
Persistent	1081 (14.6)	459 (15.7)	622 (13.9)	
Permanent	3490 (47.1)	1347 (46.0)	2143 (47.9)	
Comorbidities				
Coronary artery disease	781 (10.5)	261 (8.9)	520 (11.6)	0.115
Cardiomyopathy	634 (8.6)	348 (11.9)	286 (6.4)	<0.001
HCM	264 (3.6)	115 (3.9)	149 (3.6)	0.196
DCM	370 (5.0)	233 (8.0)	137 (3.3)	<0.001
Congenital heart disease	96 (1.3)	56 (1.9)	40 (0.9)	0.133
COPD	131 (1.8)	53 (1.8)	78 (1.7)	0.901
Hyperthyroidism	131 (1.8)	61 (2.1)	70 (1.6)	0.419
Risk factors for stroke				
Heart failure	2055 (27.7)	850 (29.0)	1205 (26.9)	<0.001
Hypertension	4477 (60.5)	0 (0.0)	4477 (100.0)	<0.001
Age (≥75 y)	2565 (34.6)	880 (30.0)	1685 (37.6)	0.002
Diabetes mellitus	1359 (18.3)	427 (14.6)	932 (20.8)	0.002
Stroke/TIA	1022 (13.8)	380 (13.0)	642 (14.3)	0.103
CHADS_2_ score				
0	1157 (15.6)	1157 (39.5)	0 (0.0)	<0.001
1	2512 (33.9)	967 (33.0)	1545 (34.5)	
≥2	3737 (50.5)	805 (27.5)	2932 (65.5)	
Mean	1.7±1.2	1.0±1.0	2.1±1.1	<0.001
CHA_2_DS_2_‐VASc score				
0	487 (6.6)	487 (16.6)	0 (0.0)	<0.001
1	1147 (15.5)	671 (22.9)	476 (10.6)	
2	1666 (22.5)	758 (25.9)	908 (20.3)	
≥3	4106 (55.4)	1013 (34.6)	3093 (69.1)	
Mean	2.8±1.6	2.0±1.4	3.3±1.5	<0.001
HAS‐BLED score, [n]	[7015]	[2766]	[4249]	
0	1117 (15.9)	603 (21.8)	514 (12.1)	<0.001
1	2689 (38.3)	1183 (42.8)	1506 (35.4)	
2	2140 (30.5)	719 (26.0)	1421 (33.4)	
≥3	1069 (15.2)	261 (9.4)	808 (19.0)	
Mean	1.5±1.0	1.3±0.9	1.6±1.0	<0.001
Systolic BP, mm Hg	126.0±16.2	120.1±14.8	129.8±15.9	<0.001
Diastolic BP, mm Hg	73.5±17.0	71.1±10.5	75.1±19.9	<0.001
Heart rate/min	72.5±13.2	72.8±12.9	72.3±13.4	0.111
Medications				
Warfarin	6404 (86.5)	2461 (84.0)	3943 (88.1)	<0.001
Dosage, mg/day	2.9±1.2	2.9±1.2	2.8±1.1	<0.001
INR				
<1.6	1670 (26.1)	674 (27.4)	996 (25.3)	0.366
1.6 to 1.99	2348 (36.7)	879 (35.7)	1469 (37.3)	
2.0 to 2.59	1854 (29.0)	704 (28.6)	1150 (29.2)	
2.6 to 2.99	363 (5.7)	143 (5.8)	220 (5.6)	
≥3.0	169 (2.6)	61 (2.5)	108 (2.7)	
Mean	1.91±0.49	1.90±0.51	1.91±0.48	0.427
TTR^b^, %	59.3±29.2	56.9±29.8	60.8±28.7	<0.001
	[n=6064]	[n=2330]	[n=3734]	
Antiplatelet	1937 (26.2)	659 (22.5)	1278 (28.5)	<0.001
Aspirin	1675 (22.6)	580 (19.8)	1095 (24.5)	<0.001
Others	433 (5.8)	129 (4.4)	304 (6.8)	<0.001
Warfarin+antiplatelet	1358 (18.3)	417 (14.2)	941 (21.0)	<0.001
Antihypertensive drugs	5354 (72.3)	1108 (37.8)	4246 (94.8)	<0.001
ARB/ACE‐I	3934 (53.1)	655 (22.4)	3279 (73.2)	<0.001
Others	3545 (47.9)	716 (24.4)	2829 (63.2)	<0.001

Data are number of patients (%) or mean±SD. ACE‐I indicates angiotensin‐converting enzyme inhibitor; AF, atrial fibrillation; ARB, angiotensin II receptor blocker; BP, blood pressure; CHADS_2_, congestive heart failure, hypertension, age ≥75 y, diabetes mellitus, and history of stroke or TIA; CHA_2_DS_2_‐VASc, additionally, vascular disease (coronary artery disease), age 65 to 74 y, and female sex; COPD, chronic obstructive pulmonary disease; DCM, dilated cardiomyopathy; HAS‐BLED, hypertension (systolic BP ≥140 mm Hg), abnormal renal/liver function, stroke, bleeding history or predisposition, labile INR (episodes of INR ≥3.5), elderly (age >65 y), drugs (use of antiplatelets)/alcohol concomitantly; HCM, hypertrophic cardiomyopathy; INR, international normalized ratio of prothrombin time; TIA, transient ischemic attack; TTR, time in therapeutic range.

a Comparison between 2 groups with and without hypertension.

b Target INR was 2.0 to 3.0 (<70 y) or 1.6 to 2.6 (≥70 y).

**Table 2:** Baseline Patient Characteristics and Medications of Each Quartile

	Quartile 1	Quartile 2	Quartile 3	Quartile 4	*P‐*Value for Trend
Range of Systolic BP, mm Hg	<116	116 to 125	126 to 135	≥136	
Number of patients	1844	1820	1765	1977	
Age, y	68.9±10.6	68.5±10.3	69.8±9.8	70.8±9.1	<0.001
Sex, male	1365 (74.0)	1301 (71.5)	1237 (70.1)	1338 (67.7)	<0.001
Type of AF					
Paroxysmal	616 (33.4)	713 (39.2)	705 (39.9)	801 (40.5)	<0.001
Persistent	286 (15.5)	268 (14.7)	264 (15.0)	263 (13.3)	
Permanent	942 (51.1)	839 (46.1)	796 (45.1)	913 (46.2)	
Comorbidities					
Coronary artery disease	197 (10.7)	198 (10.9)	204 (11.6)	182 (9.2)	0.208
Cardiomyopathy	253 (13.7)	154 (8.5)	116 (6.6)	111 (5.6)	<0.001
HCM	84 (4.6)	63 (3.5)	58 (3.3)	59 (3.0)	0.011
DCM	169 (9.2)	91 (5.0)	58 (3.3)	52 (2.6)	<0.001
Congenital heart disease	37 (2.0)	19 (1.0)	13 (0.7)	23 (1.2)	0.015
COPD	46 (2.5)	21 (1.2)	31 (1.8)	33 (1.7)	0.172
Hyperthyroidism	40 (2.2)	31 (1.7)	29 (1.6)	31 (1.6)	0.172
Risk factors for stroke					
Heart failure	683 (37.0)	495 (27.2)	445 (25.2)	432 (21.9)	<0.001
Hypertension	759 (41.2)	785 (43.1)	1144 (64.8)	1556 (78.7)	<0.001
Age (≥75 y)	587 (31.8)	630 (34.6)	603 (34.2)	745 (37.7)	<0.001
Diabetes mellitus	291 (15.8)	324 (17.8)	357 (20.2)	387 (19.6)	<0.001
Stroke/TIA	262 (14.2)	250 (13.7)	253 (14.3)	257 (13.0)	0.376
CHADS_2_ score					
0	389 (21.1)	344 (18.9)	247 (14.0)	177 (9.0)	0.135
1	609 (33.0)	586 (32.2)	621 (35.2)	696 (35.2)	
≥2	846 (45.9)	890 (48.9)	897 (50.8)	1104 (55.8)	
Mean	1.5±1.2	1.6±1.3	1.7±1.2	1.8±1.2	0.367
CHA_2_DS_2_‐VASc score					
0	170 (9.2)	148 (8.1)	106 (6.0)	63 (3.2)	<0.001
1	329 (17.8)	293 (16.1)	269 (15.2)	256 (12.9)	
2	437 (23.7)	409 (22.5)	384 (21.8)	436 (22.1)	
≥3	908 (49.2)	970 (53.3)	1006 (57.0)	1222 (61.8)	
Mean	2.6±1.6	2.7±1.6	2.9±1.6	3.0±1.5	<0.001
HAS‐BLED score [n]	[1764]	[1708]	[1664]	[1879]	
0	360 (20.4)	360 (21.1)	311 (18.7)	86 (4.6)	<0.001
1	794 (45.0)	738 (43.2)	734 (44.1)	423 (22.5)	
2	457 (25.9)	441 (25.8)	465 (27.9)	777 (23.3)	
≥3	153 (8.7)	169 (9.9)	154 (9.3)	593 (31.6)	
Mean	1.2±0.9	1.3±0.9	1.3±0.9	2.1±1.0	<0.001
Systolic BP, mm Hg	105.8±7.2	120.7±2.6	130.1±2.7	146.0±9.7	<0.001
Diastolic BP, mm Hg	65.2±19.4	71.5±9.0	76.4±21.3	80.6±11.1	<0.001
Heart rate/min	72.3±13.1	72.4±13.4	72.1±12.7	73.0±13.5	0.127
Medications					
Warfarin	1624 (88.1)	1562 (85.8)	1517 (85.9)	1701 (86.0)	0.094
Dosage, mg/day	2.8±1.2	2.9±1.2	2.9±1.2	2.9±1.2	0.004
INR					
<1.6	397 (24.4)	401 (25.7)	394 (26.0)	478 (28.1)	0.002
1.6 to 1.99	589 (36.3)	571 (36.6)	544 (35.9)	644 (37.9)	
2.0 to 2.59	487 (30.0)	478 (30.6)	441 (29.1)	448 (26.3)	
2.6 to 2.99	111 (6.8)	74 (4.7)	92 (6.1)	86 (5.1)	
≥3.0	40 (2.5)	38 (2.4)	46 (3.0)	45 (2.6)	
Mean	1.89±0.52	1.91±0.48	1.91±0.49	1.88±0.49	0.001
TTR^a^, %	58.3±28.7	59.0±29.5	60.2±29.5	59.9±29.1	0.042
	[n=1544]	[n=1488]	[n=1439]	[n=1593]	
Antiplatelet	432 (23.4)	485 (26.6)	472 (26.7)	548 (27.7)	0.004
Aspirin	376 (20.9)	431 (23.7)	409 (23.2)	449 (22.7)	0.273
Others	76 (4.1)	106 (5.8)	118 (6.7)	138 (7.0)	<0.001
Warfarin+antiplatelet	297 (16.1)	298 (16.4)	331 (18.8)	385 (19.5)	0.001
Antihypertensive drugs	1239 (67.2)	1237 (68.0)	1303 (73.8)	1575 (79.7)	<0.001
ARB/ACE‐I	867 (47.0)	882 (48.5)	958 (54.3)	1227 (62.1)	<0.001
Others	819 (44.4)	544 (46.4)	866 (49.1)	1016 (51.4)	<0.001

Data are number of patients (%) or mean±SD. ACE‐I indicates angiotensin‐converting enzyme inhibitor; AF, atrial fibrillation; ARB, angiotensin II receptor blocker; BP, blood pressure; CHADS_2_, congestive heart failure, hypertension, age ≥75 y, diabetes mellitus, and history of stroke or TIA; CHA_2_DS_2_‐VASc, additionally, vascular disease (coronary artery disease), age 65 to 74 y, and female sex; COPD, chronic obstructive pulmonary disease; DCM, dilated cardiomyopathy; HAS‐BLED, hypertension (systolic BP ≥140 mm Hg), abnormal renal/liver function, stroke, bleeding history or predisposition, labile INR (episodes of INR ≥3.5), elderly (age >65 y), drugs (use of antiplatelets)/alcohol concomitantly; HCM, hypertrophic cardiomyopathy; INR, international normalized ratio of prothrombin time; TIA, transient ischemic attack; TTR, time in therapeutic range.

a Target INR was 2.0 to 3.0 (<70 y) or 1.6 to 2.6 (≥70 y).

### Event Rates and Hypertension

**Figure 1:**
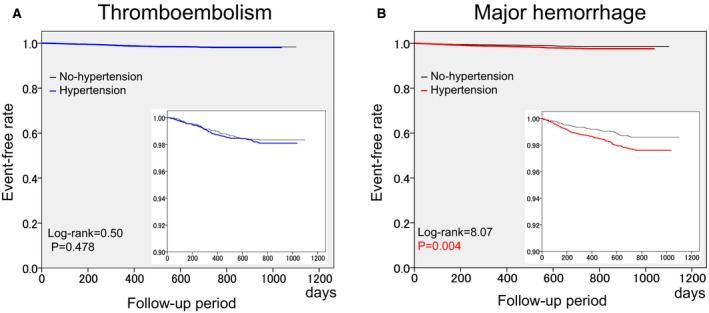
Kaplan–Meier curves of thromboembolism (A) and major hemorrhage (B). Additional boxes show the magnified event‐free curves with 10‐fold scale. *P*‐values: comparison between No‐hypertension and Hypertension groups by log‐rank test.

During the 2‐year follow‐up period, thromboembolic events occurred in 126 patients (1.7%) and major hemorrhagic events occurred in 140 patients (1.9%), including ICH in 50 patients (0.7%). The unadjusted 2‐year incidence rate of thromboembolism was similar between the 2 groups (1.8% in the Hypertension group versus 1.6% in the No‐hypertension group, *P*=0.540), whereas the rate of major hemorrhage was higher in the Hypertension group than in the No‐hypertension group (2.3% versus 1.3%, *P*=0.006) (Table 3). The Kaplan–Meier curves for thromboembolism and major hemorrhage are shown in Figure 1. There was no significant difference in the event‐free rate of thromboembolism between the 2 groups (*P*=0.478 by log‐rank test) (Figure 1A), whereas event‐free rate of major hemorrhage was significantly lower in the Hypertension group than in the No‐hypertension group (*P*=0.004 by log‐rank test) (Figure 1B). The hazard ratio (HR) of hypertension for thromboembolism was not significantly high in any model (Table 4). By contrast, the HRs of hypertension for major hemorrhage were significantly high in the crude model (Model 1) and in all adjusted models (Models 2, 3, and 4) (Table 4).

**Table 3:** Two‐Year Incidence Rates of Events

Presence of Hypertension	No‐Hypertension	Hypertension	*P‐*Value^a^
Number of patients	2929	4477	
Thromboembolism	46 (1.6%)	80 (1.8%)	0.540
Cerebral infarction	38	66	
TIA	4	5	
Systemic embolism	4	9	
Major hemorrhage	39 (1.3%)	101 (2.3%)	0.006
Intracranial	12	38	
Gastrointestinal	10	37	
Others	17	26	

Quartiles of SBP at the Time of Enrollment, mm Hg	Quartile 1 (<116)	Quartile 2 (116–125)	Quartile 3 (126–135)	Quartile 4 (≥136)	*P‐*Value for Trend
Number of patients	1844	1820	1765	1977	
Thromboembolism	32 (1.7%)	32 (1.8%)	27 (1.5%)	35 (1.8%)	0.941
Cerebral infarction	23	26	26	29	
TIA	5	3	0	1	
Systemic embolism	4	3	1	5	
Major hemorrhage	32 (1.7%)	30 (1.6%)	33 (1.9%)	45 (2.3%)	0.181
Intracranial	13	13	9	15	
Gastrointestinal	10	7	16	14	
Others	9	10	8	16	

Quartiles of SBP at the Time Closest to Event, mm Hg	Quartile 1 (<114)	Quartile 2 (114–125)	Quartile 3 (126–135)	Quartile 4 (≥136)	*P‐*Value for Trend
Number of patients^b^	1680	1869	1711	1843	
Thromboembolism	22 (1.3%)	17 (0.9%)	12 (0.7%)	66 (3.6%)	<0.001
Cerebral infarction	16	14	7	60	
TIA	4	1	2	1	
Systemic embolism	2	2	3	5	
Major hemorrhage	32 (1.9%)	25 (1.3%)	23 (1.3%)	52 (2.8%)	0.041
Intracranial	6	4	10	29	
Gastrointestinal	15	9	9	11	
Others	11	12	4	12	

Data are number of patients (%). SBP indicates systolic blood pressure; TIA, transient ischemic attack.

a Comparison between 2 groups.

b No blood pressure data at the time closest to event or at the end of follow‐up in 303 patients.

**Table 4:** Influence of Hypertension on Thromboembolism and Major Hemorrhage (Cox Proportional Hazards Model)

	Model 1		Model 2		Model 3		Model 4	
	HR (95% CI)	*P‐*Value	HR (95% CI)	*P‐*Value	HR (95% CI)	*P‐*Value	HR (95% CI)	*P‐*Value
*Thromboembolism*								
Hypertension	1.14 (0.79–1.64)	0.479	1.03 (0.72–1.49)	0.862	1.03 (0.72–1.49)	0.864	1.05 (0.73–1.52)	0.787
Heart failure	—	—	1.02 (0.70–1.50)	0.914	1.03 (0.70–1.52)	0.878	1.10 (0.75–1.63)	0.621
Age (≥75 y)	—	—	2.28 (1.60–3.29)	<0.001	2.69 (1.63–4.45)	<0.001	2.80 (1.69–4.64)	<0.001
Diabetes mellitus	—	—	1.27 (0.83–1.94)	0.271	1.25 (0.81–1.91)	0.313	1.27 (0.83–1.95)	0.275
Stroke/TIA	—	—	1.75 (1.16–2.66)	0.008	1.72 (1.13–2.61)	0.011	1.82 (1.19–2.79)	0.006
Coronary artery disease	—	—	—	—	0.96 (0.55–1.67)	0.889	0.92 (0.52–1.63)	0.771
Age (65–74 y)	—	—	—	—	1.24 (0.72–2.14)	0.435	1.31 (0.76–2.26)	0.329
Sex, female	—	—	—	—	0.78 (0.52–1.17)	0.228	0.77 (0.52–1.16)	0.210
Warfarin use	—	—	—	—	—	—	0.44 (0.28–0.68)	<0.001
Antiplatelet use	—	—	—	—	—	—	1.08 (0.71–1.64)	0.720
*Major hemorrhage*								
Hypertension	1.70 (1.17–2.46)	0.005	1.58 (1.09–2.30)	0.015	1.56 (1.08–2.27)	0.019	1.52 (1.05–2.21)	0.027
Heart failure	—	—	1.68 (1.20–2.37)	0.003	1.66 (1.18–2.32)	0.004	1.60 (1.14–2.26)	0.007
Age (≥75 y)	—	—	1.88 (1.34–2.63)	<0.001	2.61 (1.59–4.29)	<0.001	2.52 (1.53–4.14)	<0.001
Diabetes mellitus	—	—	1.26 (0.85–1.86)	0.256	1.17 (0.79–1.74)	0.441	1.14 (0.76–1.70)	0.525
Stroke/TIA	—	—	1.67 (1.12–2.49)	0.012	1.58 (1.06–2.37)	0.025	1.49 (0.99–2.23)	0.055
Coronary artery disease	—	—	—	—	1.31 (0.83–2.06)	0.240	1.16 (0.71–1.90)	0.556
Age (65–74 y)	—	—	—	—	1.52 (0.90–2.55)	0.116	1.46 (0.87–2.46)	0.154
Sex, female	—	—	—	—	0.60 (0.40–0.90)	0.013	0.61 (0.40–0.91)	0.016
Warfarin use	—	—	—	—	—	—	2.31 (1.11–4.82)	0.025
Antiplatelet use	—	—	—	—	—	—	1.32 (0.89–1.96)	0.167

Model 1: Unadjusted (crude); Model 2: Adjusted for other components of CHADS_2_ score (congestive heart failure, age ≥75 y, diabetes mellitus, and history of stroke or TIA); Model 3: Adjusted for other components of CHA_2_DS_2_‐VASc score (additionally, vascular disease [coronary artery disease], age 65–74 y, and female sex); Model 4: Adjusted for other components of CHA_2_DS_2_‐VASc score, warfarin use, and antiplatelet use. HR indicates hazard ratio; TIA, transient ischemic attack.

### Event Rates and BP at the Time of Enrollment

Systolic BP at the time of enrollment in patients who were subsequently complicated with thromboembolism or major hemorrhage (127±20 or 128±17 mm Hg) was not different from that in event‐free patients (126±16 mm Hg). Diastolic BP at the time of enrollment in patients complicated with these events during the follow‐up period (74±13 and 73±11 mm Hg) was also similar to that in event‐free patients (74±17 mm Hg).

When patients were divided into quartiles of systolic BP at the time of enrollment, HRs for thromboembolism or major hemorrhage did not differ among the quartiles in any model; this was also true for quartiles of diastolic BP at the time of enrollment (Table 5). When baseline BP values were used as a continuous variable, adjusted risk (by Model 4) of thromboembolism and major hemorrhage tended to increase slightly for every 10‐mm Hg increase in baseline systolic BP (HR 1.02, 95% CI 0.92–1.14, *P*=0.724 for thromboembolism and HR 1.09, 95% CI 0.98–1.21, *P*=0.098 for major hemorrhage), although not statistically significant either.

**Table 5:** Influence of Blood Pressure at the Time of Enrollment

	Model 1		Model 2		Model 3		Model 4	
	HR (95% CI)	*P‐*Value	HR (95% CI)	*P‐*Value	HR (95% CI)	*P‐*Value	HR (95% CI)	*P‐*Value
Thromboembolism								
Systolic BP								
Quartile 1 (<116 mm Hg)	1.00 (reference)		1.00 (reference)		1.00 (reference)		1.00 (reference)	
Quartile 2 (116–125 mm Hg)	1.00 (0.61–1.64)	0.990	0.97 (0.59–1.59)	0.905	0.98 (0.60–1.60)	0.922	0.96 (0.59–1.58)	0.881
Quartile 3 (126–135 mm Hg)	0.86 (0.52–1.44)	0.565	0.83 (0.49–1.38)	0.465	0.83 (0.50–1.39)	0.480	0.84 (0.50–1.41)	0.417
Quartile 4 (≥136 mm Hg)	1.01 (0.63–1.63)	0.968	0.96 (0.59–1.55)	0.853	0.96 (0.59–1.57)	0.878	0.86 (0.59–1.57)	0.801
Diastolic BP								
Quartile 1 (<66 mm Hg)	1.00 (reference)		1.00 (reference)		1.00 (reference)		1.00 (reference)	
Quartile 2 (66–72 mm Hg)	1.04 (0.63–1.70)	0.885	1.11 (0.68–1.83)	0.668	1.12 (0.68–1.83)	0.657	1.11 (0.68–1.82)	0.681
Quartile 3 (73–79 mm Hg)	0.96 (0.55–1.68)	0.875	1.08 (0.61–1.90)	0.795	1.09 (0.62–1.91)	0.774	1.07 (0.61–1.88)	0.821
Quartile 4 (≥80 mm Hg)	0.96 (0.60–1.55)	0.870	1.15 (0.71–1.87)	0.551	1.16 (0.72–1.88)	0.545	1.16 (0.71–1.88)	0.559
Major hemorrhage								
Systolic BP								
Quartile 1 (<116 mm Hg)	1.00 (reference)		1.00 (reference)		1.00 (reference)		1.00 (reference)	
Quartile 2 (116–125 mm Hg)	0.94 (0.57–1.55)	0.810	0.97 (0.59–1.59)	0.893	0.97 (0.59–1.61)	0.915	0.97 (0.59–1.60)	0.905
Quartile 3 (126–135 mm Hg)	1.05 (0.65–1.71)	0.836	1.09 (0.67–1.77)	0.741	1.09 (0.67–1.78)	0.726	1.10 (0.67–1.80)	0.702
Quartile 4 (≥136 mm Hg)	1.30 (0.83–2.05)	0.256	1.36 (0.86–2.15)	0.192	1.38 (0.87–2.19)	0.167	1.37 (0.87–2.18)	0.175
Diastolic BP								
Quartile 1 (<65 mm Hg)	1.00 (reference)		1.00 (reference)		1.00 (reference)		1.00 (reference)	
Quartile 2 (66–72 mm Hg)	1.33 (0.84–2.13)	0.227	1.48 (0.92–2.36)	0.103	1.50 (0.94–2.39)	0.091	1.50 (0.94–2.40)	0.089
Quartile 3 (73–79 mm Hg)	0.87 (0.49–1.54)	0.626	1.03 (0.58–1.84)	0.926	1.05 (0.59–1.88)	0.869	1.06 (0.59–1.89)	0.857
Quartile 4 (≥80 mm Hg)	1.10 (0.69–1.75)	0.680	1.39 (0.87–2.23)	0.167	1.43 (0.89–2.28)	0.140	1.42 (0.88–2.27)	0.148

Model 1: Unadjusted (crude); Model 2: Adjusted for other components of CHADS_2_ score (congestive heart failure, age ≥75 y, diabetes mellitus, and history of stroke or TIA); Model 3: Adjusted for other components of CHA_2_DS_2_‐VASc score (additionally, vascular disease [coronary artery disease], age 65–74 y, and female sex); Model 4: Adjusted for other components of CHA_2_DS_2_‐VASc score, warfarin use, and antiplatelet use. BP indicates blood pressure; HR, hazard ratio; TIA, transient ischemic attack.

### Event Rates and BP at the Time Closest to the Event

Systolic BP at the time prior to and closest to the event (mean, 10 days) in patients who were complicated with thromboembolism or major hemorrhage (140±29 or 132±30 mm Hg, respectively) was significantly higher than that at the end of follow‐up in event‐free patients (126±17 mm Hg, *P*<0.001 for each). Diastolic BP was significantly higher in patients with thromboembolism (78±15 mm Hg) than in event‐free patients (73±11 mm Hg, *P*<0.001), but did not differ between patients with major hemorrhage (75±17 mm Hg) and event‐free patients (*P*=0.108).

**Figure 2:**
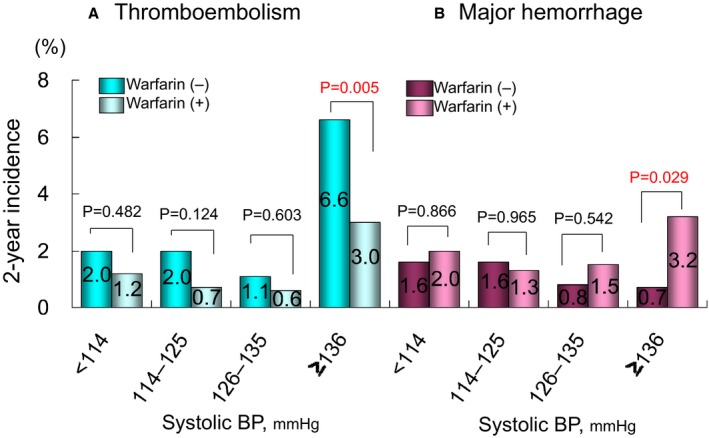
Two‐year incidence of thromboembolism (A) and major hemorrhage (B) in each quartile of blood pressure. *P*‐values: comparison between patients with and without warfarin in each quartile of blood pressure. BP, blood pressure at the time closest to the event or at the end of follow‐up.

When patients were divided into quartiles of systolic BP at the time closest to the event or at the end of follow‐up, the rate of thromboembolism was significantly lower and that of major hemorrhage was significantly higher in patients receiving warfarin of the highest quartile (Q4) (Figure 2). The ORs for thromboembolism in Q4 of systolic BP were significantly higher than those in the lowest quartile (Q1) in all models (Models 1–4); this was also true for quartiles of diastolic BP (Table 6). The OR for major hemorrhage in Q4 of systolic BP was also significantly higher than that in Q1 in adjusted models (Models 2, 3, and 4) (Table 6). No significant difference in adjusted OR for major hemorrhage was observed for diastolic BP (Table 6).

**Table 6:** Influence of Blood Pressure at the Time Closest to the Event or at the End of Follow‐Up

	Model 1		Model 2		Model 3		Model 4	
	OR (95% CI)	*P‐*Value	OR (95% CI)	*P‐*Value	OR (95% CI)	*P‐*Value	OR (95% CI)	*P‐*Value
Thromboembolism								
Systolic BP								
Quartile 1 (<114 mm Hg)	1.00 (reference)		1.00 (reference)		1.00 (reference)		1.00 (reference)	
Quartile 2 (114–125 mm Hg)	0.69 (0.37–1.31)	0.257	0.72 (0.38–1.37)	0.319	0.72 (0.38–1.37)	0.315	0.75 (0.40–1.43)	0.384
Quartile 3 (126–135 mm Hg)	0.53 (0.26–1.08)	0.081	0.56 (0.27–1.14)	0.108	0.55 (0.27–1.13)	0.103	0.56 (0.28–1.15)	0.113
Quartile 4 (≥136 mm Hg)	2.80 (1.72–4.56)	<0.001	2.87 (1.75–4.70)	<0.001	2.86 (1.74–4.69)	<0.001	2.88 (1.75–4.74)	<0.001
Diastolic BP								
Quartile 1 (<65 mm Hg)	1.00 (reference)		1.00 (reference)		1.00 (reference)		1.00 (reference)	
Quartile 2 (65–71 mm Hg)	0.66 (0.36–1.20)	0.175	0.75 (0.41–1.37)	0.348	0.75 (0.41–1.37)	0.342	0.76 (0.42–1.42)	0.411
Quartile 3 (72–79 mm Hg)	0.70 (0.38–1.29)	0.252	0.84 (0.46–1.56)	0.589	0.84 (0.46–1.56)	0.588	0.86 (0.46–1.60)	0.638
Quartile 4 (≥80 mm Hg)	1.65 (1.01–2.56)	0.046	2.04 (1.27–3.29)	0.003	2.05 (1.27–3.31)	0.003	2.09 (1.29–3.38)	0.003
Major hemorrhage								
Systolic BP								
Quartile 1 (<114 mm Hg)	1.00 (reference)		1.00 (reference)		1.00 (reference)		1.00 (reference)	
Quartile 2 (114–125 mm Hg)	0.70 (0.41–1.18)	0.182	0.76 (0.45–1.29)	0.304	0.75 (0.44–1.28)	0.291	0.74 (0.43–1.26)	0.262
Quartile 3 (126–135 mm Hg)	0.70 (0.41–1.20)	0.199	0.77 (0.44–1.32)	0.336	0.76 (0.44–1.31)	0.325	0.75 (0.44–1.30)	0.307
Quartile 4 (≥136 mm Hg)	1.50 (0.96–2.33)	0.077	1.62 (1.03–2.54)	0.038	1.62 (1.03–2.56)	0.036	1.61 (1.02–2.53)	0.041
Diastolic BP								
Quartile 1 (<65 mm Hg)	1.00 (reference)		1.00 (reference)		1.00 (reference)		1.00 (reference)	
Quartile 2 (65–71 mm Hg)	0.66 (0.36–1.20)	0.175	1.00 (0.61–1.64)	0.989	1.00 (0.61–1.63)	0.990	0.97 (0.60–1.59)	0.916
Quartile 3 (72–79 mm Hg)	0.70 (0.38–1.29)	0.252	0.95 (0.57–1.61)	0.861	0.96 (0.57–1.62)	0.879	0.95 (0.56–1.61)	0.851
Quartile 4 (≥80 mm Hg)	1.61 (1.01–2.56)	0.046	1.10 (0.69–1.77)	0.681	1.11 (0.69–1.78)	0.660	1.10 (0.68–1.76)	0.707

Model 1: Unadjusted (crude); Model 2: Adjusted for other components of CHADS_2_ score (congestive heart failure, age ≥75 y, diabetes mellitus, and history of stroke or TIA); Model 3: Adjusted for other components of CHA_2_DS_2_‐VASc score (additionally, vascular disease [coronary artery disease], age 65–74 y, and female sex); Model 4: Adjusted for other components of CHA_2_DS_2_‐VASc score, warfarin use, and antiplatelet use. BP indicates blood pressure; OR, odds ratio; TIA, transient ischemic attack.

**Figure 3:**
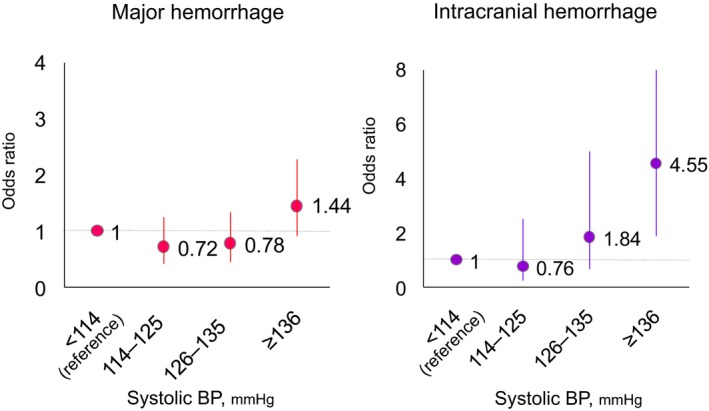
Odds ratios for major hemorrhage (A) and intracranial hemorrhage (B) in each quartile of blood pressure. Odds ratios were adjusted for other components (except hypertension) of HAS‐BLED score. See text for details. BP, blood pressure at the time closest to the event or at the end of follow‐up.

After adjustment for other components of HAS‐BLED score, the OR for major hemorrhage in Q4 of systolic BP tended to be higher than that in Q1 (OR 1.44, 95% CI 0.91–2.27, *P*=0.124). By contrast, the OR for ICH in Q4 was markedly higher than that in Q1 (OR 4.55, 95% CI 1.89–10.96, *P*=0.001) (Figure 3).

### Cutoff BP Values for Events

Areas under the curve of the receiver operating characteristic analysis for thromboembolism, major hemorrhage, and ICH were 0.663 (95% CI 0.60–0.73, *P*<0.001), 0.556 (95% CI 0.50–0.62, *P*=0.027), and 0.708 (95% CI 0.63–0.79, *P*<0.001), respectively. Cutoff BP values were determined to be 137/79 mm Hg (with a sensitivity of 55.6% and a specificity of 77.0% for systolic BP), 130/73 mm Hg (53.0% and 58.8%), and 138/78 mm Hg (57.7% and 77.6%), for thromboembolism, major hemorrhage, and ICH, respectively.

## Discussion

Although our previous reports partially revealed the influence of hypertension on event incidence,^14^, ^15^, ^16^, ^17^, ^18^, ^19^, ^20^ we confirmed in the present study that hypertension was not an independent risk factor for thromboembolism but a risk for major hemorrhage mostly in anticoagulated patients. Second, BP values at baseline were not related to the incidence of thromboembolism or major hemorrhage. By contrast, patients in the highest quartile of systolic BP (≥136 mm Hg) at the time closest to an event were characterized as high risk, with significantly higher ORs for thromboembolism, major hemorrhage, and ICH, even after adjustment for multiple confounders. Third, both the efficacy of stroke prevention and the undesirable increase in hemorrhage with warfarin were evident in the highest quartile of systolic BP of ≥136 mm Hg. Fourth, the cutoff BP values for thromboembolism, major hemorrhage, and ICH were determined to be 137/79, 130/73, and 138/78 mm Hg, respectively.

### Hypertension and Thromboembolism

Hypertension is a well‐known risk factor for stroke. In the INTERSTROKE study, a large‐scale case–control study from 22 countries, self‐reported history of hypertension was the strongest risk factor for stroke of all types (OR 2.64, 99% CI 2.26–3.08). History of hypertension also had the highest OR for ischemic stroke (OR 2.37, 99% CI 2.00–2.79) compared with any other risk factors except for AF.^25^ Increasing BP is also related to increasing stroke risk.^26^ The influence of BP values on the event rates in anticoagulated patients with AF was investigated in the combined data set of the Stroke Prevention using an ORal Thrombin Inhibitor in AF (SPORTIF) III and V trials.^27^ The HR of the highest quartile of systolic BP for stroke/systemic embolic events was 1.83 (95% CI 1.22–2.74), and the event rate for stroke/systemic embolic events increased markedly at a mean systolic BP of >140 mm Hg.^27^ In previous reports in patients with NVAF who were not receiving anticoagulation therapy, hypertension was an independent risk factor for ischemic stroke,^13^ whereas it was not in patients treated with warfarin.^14^, ^15^ Since these analyses were performed post hoc on data from prospective clinical studies, patient characteristics varied among studies.^13^, ^14^, ^15^, ^27^ In addition, since the definition of hypertension in CHADS_2_ and CHA_2_DS_2_‐VASc scores included a history of hypertension and antihypertensive drug use, measured BP values were not considered. In the present study, neither hypertension (defined as above) nor the BP value at the time of enrollment was an independent risk factor for thromboembolism. By contrast, the highest quartile of systolic BP (≥136 mm Hg) at the time closest to the event was detected as a significant risk factor for thromboembolism. Since 94.8% of patients received antihypertensive drugs and the mean BP was controlled <130 mm Hg at the time of enrollment, even in the Hypertension group, these results appear reasonable.^11^, ^12^

### Hypertension and Major Hemorrhage

Hypertension is a clear risk factor for hemorrhagic stroke in the general population.^28^ In the INTERSTROKE study, self‐reported history of hypertension was the strongest risk factor for hemorrhagic stroke (OR 3.80, 99% CI 2.96–4.78) compared with other risk factors.^25^ The effects of hypertension and BP control on event rates during antithrombotic therapy were investigated in the Bleeding with Antithrombotic Therapy (BAT) Study in Japan.^29^ An increase in BP levels during antithrombotic treatment was positively associated with the development of ICH, suggesting that adequate BP control was important for avoiding ICH.^29^ In addition, the optimal cutoff BP level to predict impending risk of ICH was determined to be ≥130/81 mm Hg.^29^ In 2 of our previous reports in patients with NVAF, hypertension was not an independent risk factor for major hemorrhage.^14^, ^15^ In the former study, which aimed to determine the impact of sex on prognosis in patients with NVAF,^14^ the adjusted OR of hypertension for major hemorrhage was comparably high to that in the present study (OR 1.48, 95% CI 0.93–2.36, *P*=0.100). However, it was not statistically significant when factors with *P*<0.25 in the antecedent univariate analysis were included as explanatory variables in the multivariate analysis. Consequently, the adopted explanatory variables differed from those in the present study. In the latter study, which aimed to assess the significance of each risk factor comprising the HAS‐BLED score in NVAF patients treated with warfarin,^15^ the adjusted HR of hypertension for major hemorrhage was not significantly high (HR 1.15, 95% CI 0.73–1.74, *P*=0.54), when the definition of hypertension was a systolic BP ≥140 mm Hg at the time of enrollment. By contrast, hypertension was detected as a significant risk factor for major hemorrhage in the present study, even after adjustment for other components of CHA_2_DS_2_‐VASc score, warfarin use, and antiplatelet use (Table 4). Hypertension, when including a history of hypertension in the definition, may have a larger contribution to the incidence of hemorrhagic events than to thromboembolism. By contrast, BP value at the time of enrollment was not a significant risk factor for major hemorrhage; however, systolic BP value at the time prior to and closest to the event was an independent risk factor for both major hemorrhage and thromboembolism, even in multiple adjusted models (Table 4). BP values at the time closest to the event would be more important for predicting both events than BP values at the time of enrollment.

In addition, we assessed the ORs of BP quartiles for major hemorrhage and ICH after adjustment for other components of the HAS‐BLED score.^15^, ^18^ This score contains warfarin and antiplatelet use, both of which are included in Model 4 of the present study. Although the OR for major hemorrhage in the highest quartile of systolic BP was not significant, the OR for ICH in the highest quartile of systolic BP at the time closest to the event was markedly higher compared with that in the lowest quartile, even after adjustment for the component of HAS‐BLED score (Figure 3). A systolic BP of ≥136 mm Hg would be a strong risk factor for ICH.

### Optimal Cutoff BP Values for Events

The present study has provided the cutoff BP values to predict impending risk of events. The accuracy of the receiver operating characteristic curves for thromboembolism and major hemorrhage based on areas under the curve was fair; the cutoff systolic BP values for these events (137 and 130 mm Hg, respectively) in the present analyses were comparable to those in previous recommendations.^29^, ^30^ The cutoff systolic BP for ICH in patients with NVAF in the present study (138 mm Hg) was higher than that in the BAT study (130 mm Hg).^29^ Patients in the BAT study were taking oral antithrombotic agents for the secondary prevention of cardiovascular or cerebrovascular diseases, regardless of AF,^29^ and therefore would be at higher risk for ICH than those in the present study.

## Limitations

The present study had several limitations. First, this study was a post hoc analysis of data from the J‐RHYTHM Registry^3^, ^16^ and was therefore hypothesis‐generating in nature. Second, the registry was established in only 158 selected institutions in Japan and most of the participating physicians specialized in cardiology and in the management of cardiac arrhythmias. Therefore, these results may not be generalizable to the overall Japanese population with NVAF. Third, the method of BP measurement was not standardized. BP values were obtained by the auscultatory method or an automated sphygmomanometer, as appropriate for daily clinical practice in each institution. Although visit‐to‐visit variability in BP is a known risk factor for stroke,^31^ it was not considered in the present analysis. Instead, BP values at the time of enrollment and the time prior to and closest to the event were selected for the analysis. Changes in antihypertensive drugs and dosages during the follow‐up period were not considered in the analysis. Fourth, the HAS‐BLED score was missing in 391 patients with NVAF; these patients were excluded from Figure 3. In these 391 patients, thromboembolism occurred in 6 and major hemorrhage occurred in 9 patients, but no ICH events were observed.

## Conclusions

A systolic BP of ≥136 mm Hg at the time closest to the event was an independent risk for thromboembolism and major hemorrhage. BP control appeared to be more important than a history of hypertension and baseline BP values at preventing thromboembolism and major hemorrhage in patients with NVAF.

## Appendix

The following persons participated in the J‐RHYTHM Registry: Executive Committee: H. Inoue, K. Okumura, H. Atarashi, and T. Yamashita. Local Executive Committee: M. Sakurai, Y. Kawamura (Hokkaido); K. Okumura, I. Kubota (Tohoku); Y. Kaneko, K. Matsumoto (North Kanto); S. Ogawa, H. Atarashi, T. Yamashita (South Kanto); H. Inoue, Y. Aizawa (Hokuetsu); I. Kodama, E. Watanabe (Chubu); Y. Koretsune, Y. Okuyama (Kansai); A. Shimizu, O. Igawa (Chugoku); S. Bando, M. Fukatani (Shikoku); T. Saikawa, A. Chishaki (Kyushu). Statistical Advisor: H. Origasa. Participating Investigators: N. Kato, K. Kanda, J. Kato, H. Obata, M. Aoki, H. Honda (Hokkaido); Y. Konta, T. Hatayama, Y. Abe, K. Terata, T. Yagi, A. Ishida, T. Komatsu, H. Tachibana, H. Suzuki, Y. Kamiyama, T. Watanabe, M. Oguma, M. Itoh, O. Hirono, Y. Tsunoda, K. Ikeda, T. Kanaya, K. Sakurai, H. Sukekawa, S. Nakada (Tohoku); T. Itoh, S. Tange, M. Manita, M. Ohta, H. Eguma, R. Kato, Y. Endo, T. Ogino, M. Yamazaki, H. Kanki, M. Uchida, S. Miyanaga, K. Shibayama, N. Toratani, T. Kojima, M. Ichikawa, M. Saito, Y. Umeda, T. Sawanobori, H. Sohara, S. Okubo, T. Okubo, T. Tokunaga, O. Kuboyama, H. Ito, Y. Kitahara (North Kanto); K. Sagara, T. Satoh, E. Kodani, K. Sugi, Y. Kobayashi, Y. Higashi, T. Katoh, Y. Hirayama, N. Matsumoto, M. Takano, T. Ikeda, S. Yusu, S. Niwano, Y. Nakazato, Y. Kawano, M. Sumiyoshi, N. Hagiwara, K. Murasaki, H. Mitamura, S. Nakagawa, K. Okishige, K. Azegami, H. Aoyagi, K. Sugiyama, M. Nishizaki, N. Yamawake, I. Watanabe, K. Ohkubo, H. Sakurada, S. Fukamizu, M. Suzuki, W. Nagahori, T. Nakamura, Y. Murakawa, N. Hayami, K. Yoshioka, M. Amino, K. Hirao, A. Yagishita, K. Ajiki, K. Fujiu, Y. Imai, A. Yamashina, T. Ishiyama (South Kanto); M. Sakabe, K. Nishida, H. Asanoi, H. Ueno, J. D. Lee, Y. Mitsuke, H. Furushima, K. Ebe, M. Tagawa, M. Sato, M. Morikawa (Hokuetsu); K. Yamashiro, K. Takami, T. Ozawa, M. Watarai, M. Yamauchi, H. Kamiya, H. Hirayama, Y. Yoshida, T. Murohara, Y. Inden, H. Osanai, N. Ohte, T. Goto, I. Morishima, T. Yamamoto, E. Fujii, M. Senga, H. Hayashi, T. Urushida, Y. Takada, R. Kato, N. Tsuboi, T. Noda, T. Hirose, T. Onodera, S. Kageyama, T. Osaka, T. Tomita, K. Shimada, M. Nomura, H. Izawa, A. Sugiura, T. Arakawa, K. Kimura (Chubu); T. Mine, T. Makita, H. Mizuno, A. Kobori, T. Haruna, M. Takagi, T. Watanabe, N. Tanaka, H. Shimizu, T. Kurita, K. Motoki, N. Takeda, Y. Kijima, M. Ito, A. Nakata, Y. Ueda, A. Hirata, S. Kamakura, K. Satomi, T. Noda, Y. Yamada (Kansai); Y. Yoshiga, H. Ogawa, M. Kimura, T. Hayano, T. Kinbara, H. Tatsuno, M. Harada, K. F. Kusano, M. Adachi, A. Yano, M. Sawaguchi, J. Yamasaki, T. Matsuura, Y. Tanaka, H. Moritani, T. Maki, S. Okada, M. Takechi, T. Hamada (Chugoku); A. Nishikado, Y. Takagi, I. Matsumoto, T. Yamamoto, T. Soeki, Y. Doi, M. Okawa, H. Seo, S. Kitamura, K. Yamamoto, M. Akizawa, N. Kaname (Shikoku); S. Ando, S. Narita, T. Nakamura, T. Inou, Y. Fukuizumi, K. Saku, M. Ogawa, Y. Urabe, M. Ikeuchi, S. Harada, H. Yamabe, Y. Imamura, Y. Yamanouchi, K. Sadamatsu, K. Yoshida, T. Kubota, N. Takahashi, N. Makino, Y. Higuchi, T. Ooie, T. Iwao, K. Kitamura, T. Imamura, K. Maemura, N. Komiya, M. Hayano, H. Yoshida, K. Yamashiro, K. Kumagai (Kyushu).

## Sources of Funding

The J‐RHYTHM Registry is registered at University hospital Medicine Information Network (UMIN) Clinical Trials Registry (UMIN000001569) and was supported by a grant from the Japan Heart Foundation (12080025). This research was partially supported by the Practical Research Project for Life‐Style related Diseases including Cardiovascular Diseases and Diabetes Mellitus from Japan Agency for Medical Research and Development (AMED) (15656344).

## Disclosures

Dr Atarashi received research funding from Boehringer Ingelheim, and remuneration from Bayer Healthcare, Boehringer Ingelheim, and Daiichi‐Sankyo; Dr Inoue received remuneration from Daiichi‐Sankyo, Bayer Healthcare, and Bristol‐Myers Squibb; Dr Okumura received research funding from Boehringer Ingelheim and Daiichi‐Sankyo and remuneration from Boehringer Ingelheim, Bayer Healthcare, Daiichi‐Sankyo, and Pfizer; Dr Yamashita received research funding from Daiichi‐Sankyo, Bayer Healthcare, Tanabe‐Mitsubishi, Ono Pharmaceutical, and Bristol‐Myers Squibb and remuneration from Daiichi‐Sankyo, Pfizer, Bayer Healthcare, Bristol‐Myers Squibb, Boehringer Ingelheim, Eisai, and Ono Pharmaceutical; Dr Origasa received remuneration from Daiichi‐Sankyo and Bayer Healthcare.
